# Beyond xMOOCs in healthcare education: study of the feasibility in integrating virtual patient systems and MOOC platforms

**DOI:** 10.7717/peerj.672

**Published:** 2014-11-13

**Authors:** Natalia Stathakarou, Nabil Zary, Andrzej A. Kononowicz

**Affiliations:** 1 Department of Learning Informatics Management and Ethics, Karolinska Institutet, Stockholm, Sweden; 2 Department of Bioinformatics and Telemedicine, Jagiellonian University Medical College, Kraków, Poland

**Keywords:** Virtual patients, Healthcare education, e-learning, Massive open online courses, Integration

## Abstract

**Background.** Massive Open Online Courses (MOOCs) are an emerging trend in online learning. However, their technology is not yet completely adjusted to the needs of healthcare education. Integration of Virtual Patients within MOOCs to increase interactivity and foster clinical reasoning skills training, has been discussed in the past, but not verified by a practical implementation.

**Objective.** To investigate the technical feasibility of integrating MOOCs with Virtual Patients for the purpose of enabling further research into the potential pedagogical benefits of this approach.

**Methods.** We selected OpenEdx and Open Labyrinth as representative constituents of a MOOC platform and Virtual Patient system integration. Based upon our prior experience we selected the most fundamental technical requirement to address. Grounded in the available literature we identified an e-learning standard to guide the integration. We attempted to demonstrate the feasibility of the integration by designing a “proof-of-concept” prototype. The resulting pilot implementation was subject of verification by two test cases.

**Results.** A Single Sign-On mechanism connecting Open Labyrinth with OpenEdx and based on the IMS LTI standard was successfully implemented and verified.

**Conclusion.** We investigated the technical perspective of integrating Virtual Patients with MOOCs. By addressing this crucial technical requirement we set a base for future research on the educational benefits of using virtual patients in MOOCs. This provides new opportunities for integrating specialized software in healthcare education at massive scale.

## Background

Significant changes in the healthcare sector and increased learning expectations require contemporary healthcare education to reform and respond to the new challenges. In particular, demographic changes and the growing population demand an increase in trainees’ required competencies and call for better training of higher order skills (Frenk et al., 2010). The learning opportunities provided to medical students for observing the treatment process in hospitals are diminished (Kononowicz & Hege, 2010). At the same time, the limited access to public medical education, the technological innovations entering the field of healthcare (Frenk et al., 2010) and the rapid expansion of new medical knowledge generated by clinical research (Marya & Zilberberg, 2011) highlight the need for massive and continuing healthcare education.

### Massive open online courses

In the evolving process of online education, Virtual Learning Environments (VLEs) have undergone considerable changes (Masters, 2009). Currently VLEs are being prepared to be used at large scale in Massive Open Online Courses (MOOCs). MOOCs are a new form of learning activities on the Internet providing free access to elite universities’ courses for an unlimited number of participants (Yuan & Powell, 2013).

MOOC platforms are currently at different levels of development but share common features. The content is delivered online by a set of tools forming the infrastructure of the course (Kay et al., 2013). They are decentralized, networked and based on cloud computing technologies (Sonwalkar, 2013). A central VLE unit for MOOCs has diminished in importance and constitutes usually only one component in the MOOC’s network. It is used mainly for administrative purposes, such as students’ registrations, and for hosting of discussion boards. The remaining parts are external tools where the students’ activities concentrate. This includes in particular web applications, which are used to host lectures in forms of videos, support the learners’ interaction, collaboration, evaluation and self-assessment (Yousef et al., 2014). The course instructions consist of descriptions with links to tools and are distributed to the participants in the form of newsletters (Masters, 2009).

The first MOOCs, known as cMOOCs, explored new pedagogies beyond the traditional classroom context and allowed the learners to construct self-organized and social learning processes based on interaction. The learners’ participation generates the content of the course, while the level and type of their participation depends on each individual learner (Masters, 2009). Many of the massive courses that followed, however, are an extension of the lectured-based pedagogical models practiced in institutions (Yuan & Powell, 2013). Their name, xMOOCs, is associated with the non-profit platform edX, launched by Harvard and Massachusetts Institute of Technology, to provide online courses to mass audience (Grünewald et al., 2013).

Besides their technical innovation, xMOOCs are based on the theoretical presentation of the learning context, supplemented by interactive tasks and discussion boards activities: “cMOOCs focus on knowledge creation and generation whereas xMOOCs focus on knowledge duplication” (Siemens, 2012). xMOOCs’ learning objectives are predefined by the courses’ instructors, while the participants’ communication is limited (Yousef et al., 2014). For this reason, xMOOCs are criticized for replicating traditional pedagogical models.

This first differentiation of MOOC types resulted in a wave of new terms to label variants of Open Online Courses in respect to scale, pedagogical model or target audience. Vocational Open Online Courses are delivered for wide but targeted audiences with the aim to foster career progression and leverage specific work-related skills (Clark, 2014). Small Private Online Courses (SPOCS) support flipped classroom learning; the online lectures are delivered to a limited number of participants of a college class. The instructors may use the actual class time to provide the remaining, more student-tailored, learning experience (Fox, 2013).

The massiveness of the MOOC audience and the diversity in their background implies challenges regarding the development and integration of open services, the scalability in the infrastructure and the automation of tasks. At the same time, the integration of open services may support the educational process and increase the interactivity of the learning environment (Claros et al., 2013).

The medical and healthcare education community is actively investigating the potential for adapting MOOCs and other forms of online education to fit their own needs, predicting that online medical courses will be commonplace within the next five years (Harder, 2013). MOOCs may support undergraduate, graduate and continuing medical education (CME) by having the potential to address some of the current challenges of the healthcare education (Mehta et al., 2013). They assert, however, that lecture-based courses form only part of the educational experience which should be provided. In particular, the technological infrastructure of MOOCs may foster learner communication and interaction to some degree, but not necessarily to the extent that healthcare education requires (Harder, 2013).

### Virtual patients

Virtual patients (VPs) are defined as “interactive computer simulations of real-life clinical scenarios for the purpose of healthcare and medical training, education or assessment” (Ellaway et al., 2006). This definition distinguishes the VPs from devices, human standardized patients, part task trainers and high fidelity manikins (Talbot et al., 2012). VPs have become an established tool in healthcare teaching and assessment. In particular, VPs are suggested as the key technology that can develop the fundamental skill of clinical reasoning amongst students, allowing students to develop these skills to a similar level as that achieved whilst training with real patients (Cook & Triola, 2009).

By emulating the role of the healthcare professional, the learner is provided with training opportunities to identify relevant information from a set of anonymous patient-related data, conduct physical exams, laboratory tests and make diagnostic and therapeutic decisions (AAMC Institute for Improving Medical Education, 2007) without any real world repercussions. VPs are reported to be a response to some of the current challenges in medical education (Round, Conradi & Poulton, 2009) such as the limited learning opportunities for observing the treatment process. Moreover, VPs “fill gaps in clerkships by exposing students to diseases that they would not otherwise experience because of short clinical rotations and limited ambulatory care experiences” (Huang, Reynolds & Candler, 2007).

Although there is evidence to support the effectiveness of training clinical reasoning skills using VPs (Consorti et al., 2012), they “play only one part in the development of skilled health professionals” and coordination with other instructional activities is suggested (Cook & Triola, 2009). Positive effects have been reported when VPs are used as an additional resource or as an alternative to traditional methods (Consorti et al., 2012).

A significant barrier that medical faculties often encounter in integrating VPs in their curriculum is the timely, costly and complex process of producing and authoring VPs. VP systems have been extended in the past in order to support content transfer and by that to enable the technical sharing of the VP cases among institutions. That was achieved by applying the MedBiquitous Virtual Patient standard (MVP) (Hege et al., 2009).

Besides the technical sharing of the VPs, the cases require meeting ethnic, language and socio-economic aspects of the institutions in which are used (Fors et al., 2009). The process of adapting the VP cases to meet these requirements is known as “repurposing”. The electronic Virtual Patient project (eViP) was an initiative in which nine European institutions and MedBiquitous collaborated to create a repurposed and enriched collection of VPs publicly available (Hege et al., 2009; Zary et al., 2009).

### Problem description

Whilst the features of MOOCs offer the potential to enhance the learning process by promoting interactivity and self-directed learning, their contemporary form is limited to passive transmission of knowledge, based on the presentation of videos. Moreover, from the technical and pedagogical perspective their application in the healthcare education is still in the early stages of investigation (Liyanagunawardena & Williams, 2014).

In order to successfully deliver e-learning resources in a healthcare context an important factor to consider is accessibility (Childs et al., 2005); VPs may be integrated with VLEs to meet accessibility requirements. Requirements and integration strategies before the MOOC era were proposed and demonstrated in past studies (Kononowicz et al., 2010). One of the requirements is a Single Sign-On mechanism (SSO) for institution-wide access to e-learning tools: “it is a significant drawback of the current VP implementations to require separate authentication mechanisms” (Kononowicz et al., 2010). Integration can be achieved partially or fully by applying e–learning standards. In one of the former studies “a point-to-point connection was implemented from the VLE Moodle to the LMU’s VP system CASUS” using the SCORM/AICC-HACP standard (Kononowicz et al., 2010). From our current perspective the reported technical implementation was based on outdated standards and was adjusted to the previous generation of VLE and VP systems, without taking into account the technology of MOOC platforms.

The educational possibilities of extending MOOCs with VPs, with the goal of fostering medical competencies such as clinical reasoning, were discussed by us theoretically in the past (Stathakarou, Zary & Kononowicz, 2014). In particular, we proposed three educational scenarios taking advantage of VP features, augmented by the distributed knowledge base provided by participants and the mass customization features of MOOCs. However, the technical feasibility for implementing the suggested use cases has not yet been examined. To the best of our knowledge there are no previously reported technical attempts of using virtual patients at large scale as part of MOOCs.

### Study objectives

This study aims to investigate how to technically integrate virtual patient systems with MOOC platforms. Such knowledge will inform the feasibility of further educational research and evaluation of the potential educational benefits. Bearing in mind the vast topic we predict that the implementation will follow an iterative step-wise approach starting with the most fundamental requirements. The objective of the study is to select such a technical requirement and implement it as a prototype.

## Material and Methods

We designed our research as a feasibility study to verify the idea of integrating virtual patient systems and MOOC platforms. This includes building a “proof-of-concept” prototype to evaluate if the system design can be implemented and will provide reasonable output (Friedman & Whatt, 1997). In this section we describe preparations for an implementation and our approach to verifying the resulting prototype.

### Selected requirement to be addressed

From the previously established technical requirements (Stathakarou, Zary & Kononowicz, 2014) a central one was selected for implementation: a transparent authentication of the learner enabled by an identity management mechanism. The feasibility study evaluates a type of Single Sign-On technique integrating a virtual patient system and a MOOC platform that has not been demonstrated previously. It enables a user logged into the MOOC platform to access VP cases without requiring a repeated manual entry of the credentials. From the user perspective, the moment of entering a second system is unnoticeable, which saves time and improves user satisfaction with the learning experience. This feature is useful both for instructors and learners of a MOOC course, and is a prerequisite for the implementation of more advanced functions.

### Study material

The study is conducted on the example of two actual systems: the OpenEdx MOOC platform and Open Labyrinth virtual patient system Openlabyrinth.ca.

The edX initiative was launched by Massachusetts Institute of Technology (MIT) and Harvard and offers not-for-profit online and in the classroom education. The edX platform is hosting MOOCs of global partner institutions and organizations. The open-source release of the edX platform is named OpenEdx. The selected MOOC platform is comparable with other available ones such as Coursera or Udacity, therefore the choice of platform does not influence the generalizability of the study. Access to the authoring system of the edX platform, edX Studio, was enabled by Karolinska Institutet becoming a member of the edX initiative in 2013.

The Open Labyrinth virtual patient system is a project developed and maintained by an international consortium of universities. Open Labyrinth is a web application for authoring and displaying VP cases. It is currently the most advanced, freely available, open-source virtual patient system. Because of its conformance to the ANSI accredited MedBiquitous Virtual Patient standard (Hege et al., 2009; Zary et al., 2009) it may be regarded as representative for the class of virtual patient systems.

### Study protocol

**Figure 1:**
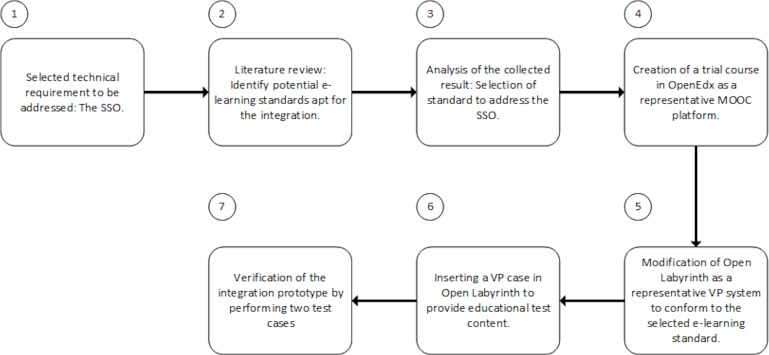
Study protocol.

In order to explore the viable ways of integrating a VP system into a MOOC platform a literature review was conducted to identify the potential standards which were suitable for the integration. The collected results were examined to address the selected technical requirement. To construct the prototype a trial course was created in the OpenEdx platform. Open Labyrinth was modified accordingly to conform to the selected e-learning standard. A VP case was imported into Open Labyrinth for the purpose of providing test educational content. Finally, two test cases were performed to verify the implementation. The process followed in this study is visualized in Fig. 1.

### Data collection

To identify the standards apt for implementing the integration, we reviewed the databases of Scopus, ERIC and PubMed. The review was chronologically limited to publications of the range 2008–2014, to discard outdated technologies. The review was performed using the following queries:

• Integration AND “Virtual Patient^∗^”
• Integration AND MOOC^∗^
• e-learning AND standard AND integration

We identified two research papers summarizing the most relevant outcomes from the e-learning standardization efforts, as well as a taxonomy of the interoperability types (Anido-Rifón et al., 2014; Del Blanco et al., 2013)

From the e-learning standards which had the potential to support the integration of the two environments in terms of implementing the SSO, and giving consideration to the requirements of the OpenEdx and Open Labyrinth systems, we selected the IMS LTI standard (IMS Global Consortium, 2014): a framework for integrating e-learning tools and content into VLEs. According to the terminology followed in the specification of LTI, the VLE is referred as a “Tool Consumer”, meaning that it “consumes” the external tool to be integrated, while the tool is named “Tool Provider”. The Basic LTI (BLTI), a subset of the overall functionality of the LTI, establishes a one-launch mechanism from the consumer to the provider.

Both EdX and OpenEdx platforms conform to the BLTI standard, meaning that they can act as a tool consumer. Open Labyrinth however required adjustments to function as a tool provider. The BLTI makes use of the OAuth protocol signing approach (OAuth) to secure the message interactions between the consumer and the provider, which requires a set of credentials: a key and a secret.

### Test environment

Our implementation focused on the SSO mechanism connecting the two selected systems: OpenEdx and Open Labyrinth. For the purpose of prototyping, we created a sample course in OpenEdx. The course was not publicly released and was used only for the purpose of this study.

Open Labyrinth was set up on a virtual LAMP server, launched through Amazon Elastic Compute Cloud (EC2) in order to prepare and finalize the adjustments required for the integration (Amazon EC2). The advantage of this solution is that EC2 includes an auto-scale option which allows the instance to meet potential increased load.

In order to provide educational content in the course for test purposes we manually imported a VP case from the eViP project repository. The selected case refers to bronchogenic carcinoma which is an important topic in medical education, since it is the most common cause of cancer-related deaths worldwide (Ferlay et al., 2010).

### Verification using test cases

We verified the technical implementation by performing test cases. The test cases were designed in order to evaluate the system’s response to different input requests. The following distinct test cases were developed to test the transparent authentication mechanism:

• An instructor logged into the OpenEdx platform is automatically authorized in Open Labyrinth to access and edit an existing VP case, or author a new one.
• A learner logged into the OpenEdx platform is automatically authorized in Open Labyrinth to access the VP case.

In particular, the logged in trial OpenEdx course users, should be able to get authorized in Open Labyrinth and access the VP case through the platform, without requiring a repeated entry of their credentials.

## Results

This section describes in detail the resulting integration prototype. The SSO mechanism was implemented following the BLTI standard and evaluated by two test cases.

### Modifications to the Open Labyrinth VP system to act as a tool provider

The implementation required modifying Open Labyrinth in order to function as a tool provider integrated in the OpenEdx platform. The OpenEdx user, by selecting the Open Labyrinth link to access the content, issues a BLTI launch request, where a HTTP POST message transmits a set of data elements required to authorize the user. This is imposed both by the oAuth standard and the LTI specification.

In order to implement the BLTI interface we programmed the elementary framework classes of Basic LTI (files blti.php and oauth.php) as indicated by the IMS-LTI specification. We also created two new files named user-handler.php and database.php and we modified the index.php page of Open Labyrinth. In the database of Open Labyrinth we added a new entry to maintain the credentials (key, secret).

Then we modified the landing page of Open Labyrinth to intercept the data that are passed on by a BLTI launch request. The code in index.php file receives the data and transmits them to the BLTI and OAuth classes in order to be verified. The BLTI class firstly confirms that a minimum set of values to meet the protocol requirements has been received and then, using the obtained key looks up the corresponding value of the secret in the database.

Next, by using the OAuth signing mechanism the signature is re-computed and compared with the one received from the LTI launch request to verify the credentials of the sender. The set of values received are additionally checked for their appropriateness according to the protocol. If the values are not appropriate the BLTI class will reject the connection. The connection and queries to the database are managed through the homonymous file. If the signatures’ comparison is successful, the user-handler class is called to manage the user.

On receiving the user’s details by the blti.php file, the user-handler class first looks in the database to identify whether the user’s entry already exists and if not, creates a new one to register the user. Then, by using the log-in function, it allows access to the user and returns to the index class. The user-handler class matches the user’s role acquired by the BLTI class to the corresponding one in Open Labyrinth in order to provide the appropriate user rights. Moreover, it includes the function to encrypt the user’s password that will be maintained in the Open Labyrinth’s database.

### Connecting OpenEdx MOOC platform and Open Labyrinth using LTI

**Figure 2:**
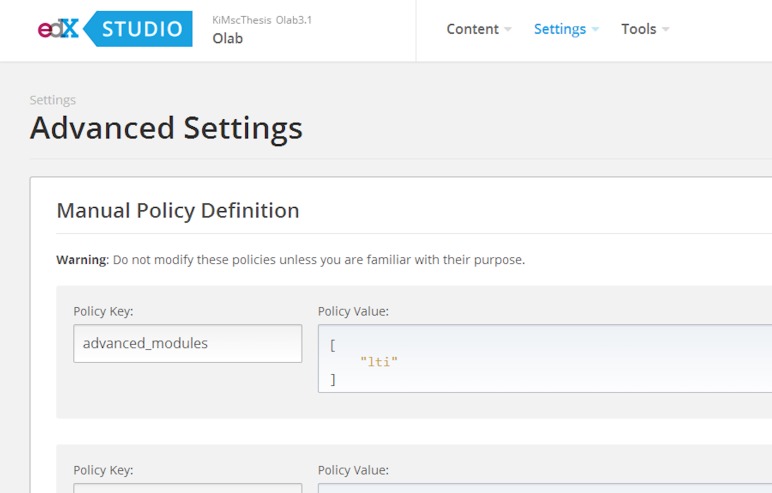
Setting the LTI module within OpenEdx to register values of lti_id, key and secret.

**Figure 3:**
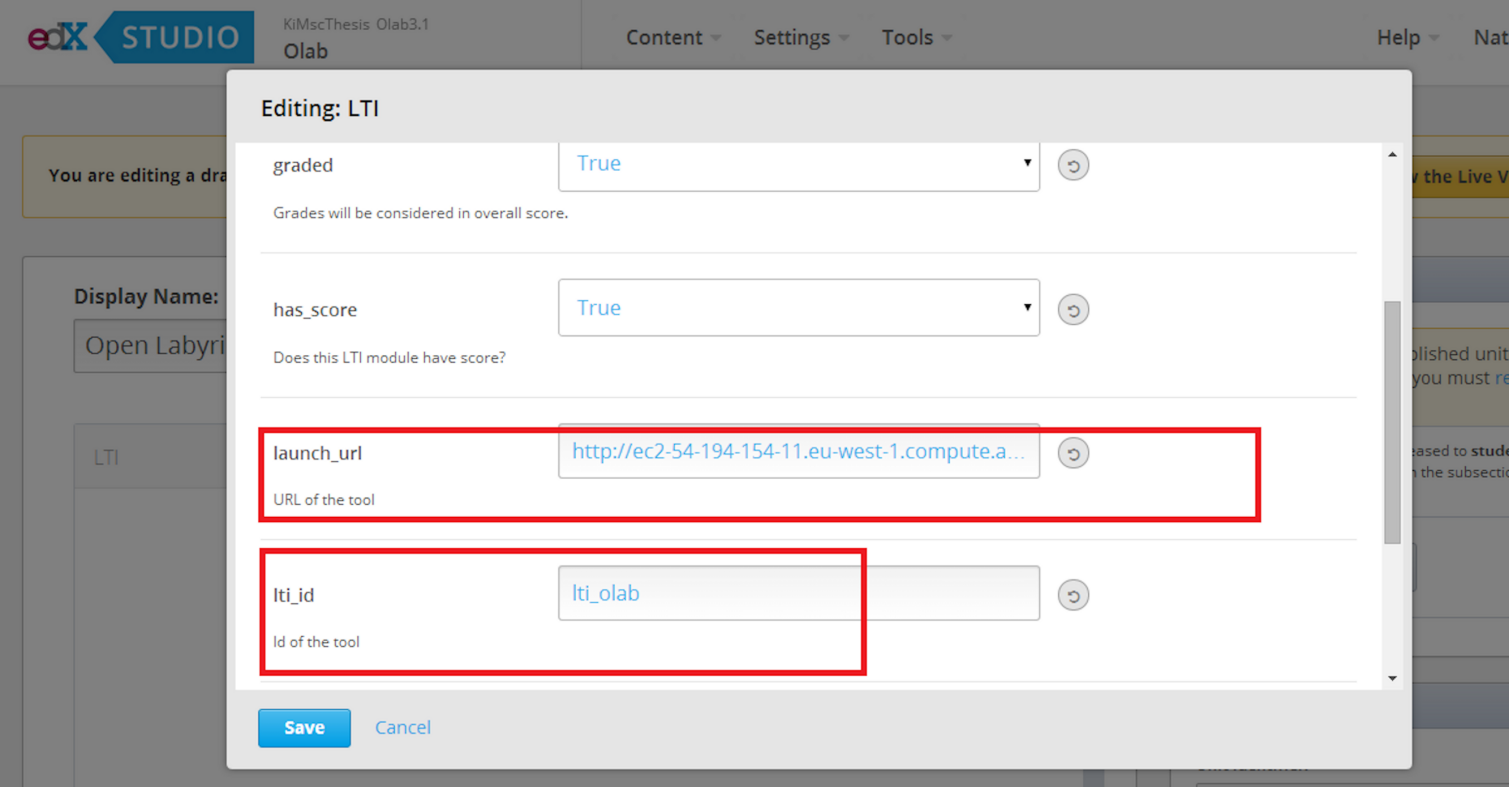
Creating a LTI component within the course, pointing at the Open Labyrinth’s server IP and including the lti_id value.

The process for linking the adjusted Open Labyrinth to the OpenEdx platform can be synopsized in the following steps:

• We created a pilot course in OpenEdx (Fig. 1).
• We added the LTI module in the advanced setting of the course, by registering customized values for the lti_id, key and secret. The lti_id is an extra parameter included in OpenEdx that can maintain any value; its role is to label the integrated component and bind the values of key and secret (Fig. 2).
• We added an LTI component within the pilot course, including the lti_id parameter and a link to the modified Open Labyrinth (Fig. 3).

### Verification of the technical implementation

In Tables 1 and 2 we present the test cases used to verify the pilot implementation. The selection of the test cases was informed by the aims of the implementation to provide a transparent authentication and access rights mechanism for the users with the appropriate credentials.

**Table 1:** Test Case 1—an instructor gets authorized in Open Labyrinth in OpenEdx.

Test Case id	1
Objective	An instructor logged into the OpenEdx platform is automatically authorized in Open Labyrinth to access and edit an existing VP case, or author a new one
Result	Successful
Comment	Open Labyrinth authorizes the instructor, provides access to the content and authoring rights to the VP cases as a result of conforming to the BLTI standard

**Table 2:** Test Case 2—a learner gets authorized in Open Labyrinth in OpenEdx.

Test Case id	2
Objective	A learner logged into the OpenEdx platform is automatically authorized in Open Labyrinth to access the VP case
Result	Successful
Comment	Open Labyrinth authorizes the learner and allows access to the VP case as a result of conforming to the BLTI standard

**Figure 4:**
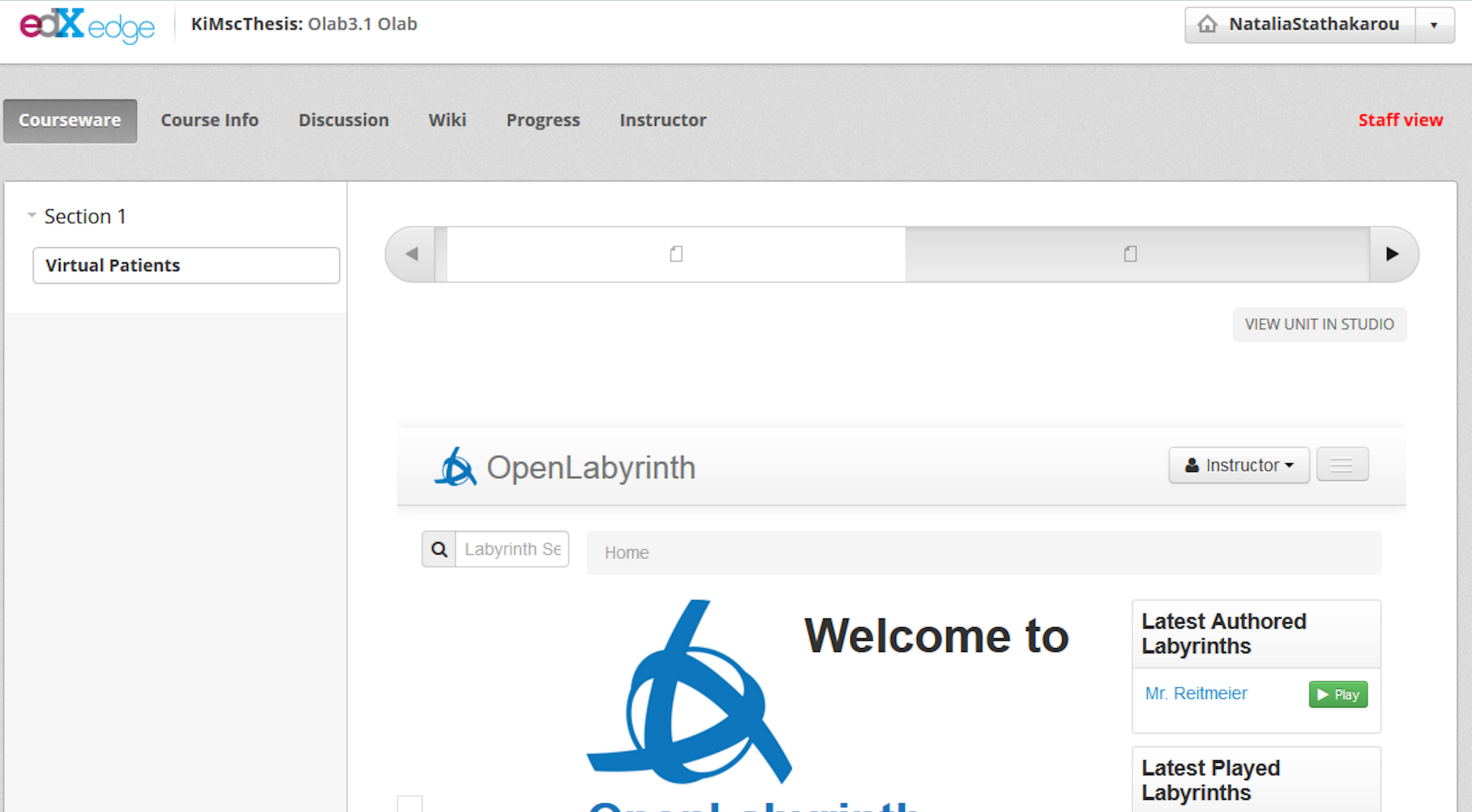
An instructor is authorized in Open Labyrinth to edit the learning content.

Figure 4 depicts the integrated Open Labyrinth in the OpenEdx platform from the perspective of the instructor after the automatic authorization step. The instructor is provided with the user rights imposed by the corresponding administrator’s role of Open Labyrinth. By that, the instructor may access, edit or delete the content in Open Labyrinth or author a new VP case.

**Figure 5:**
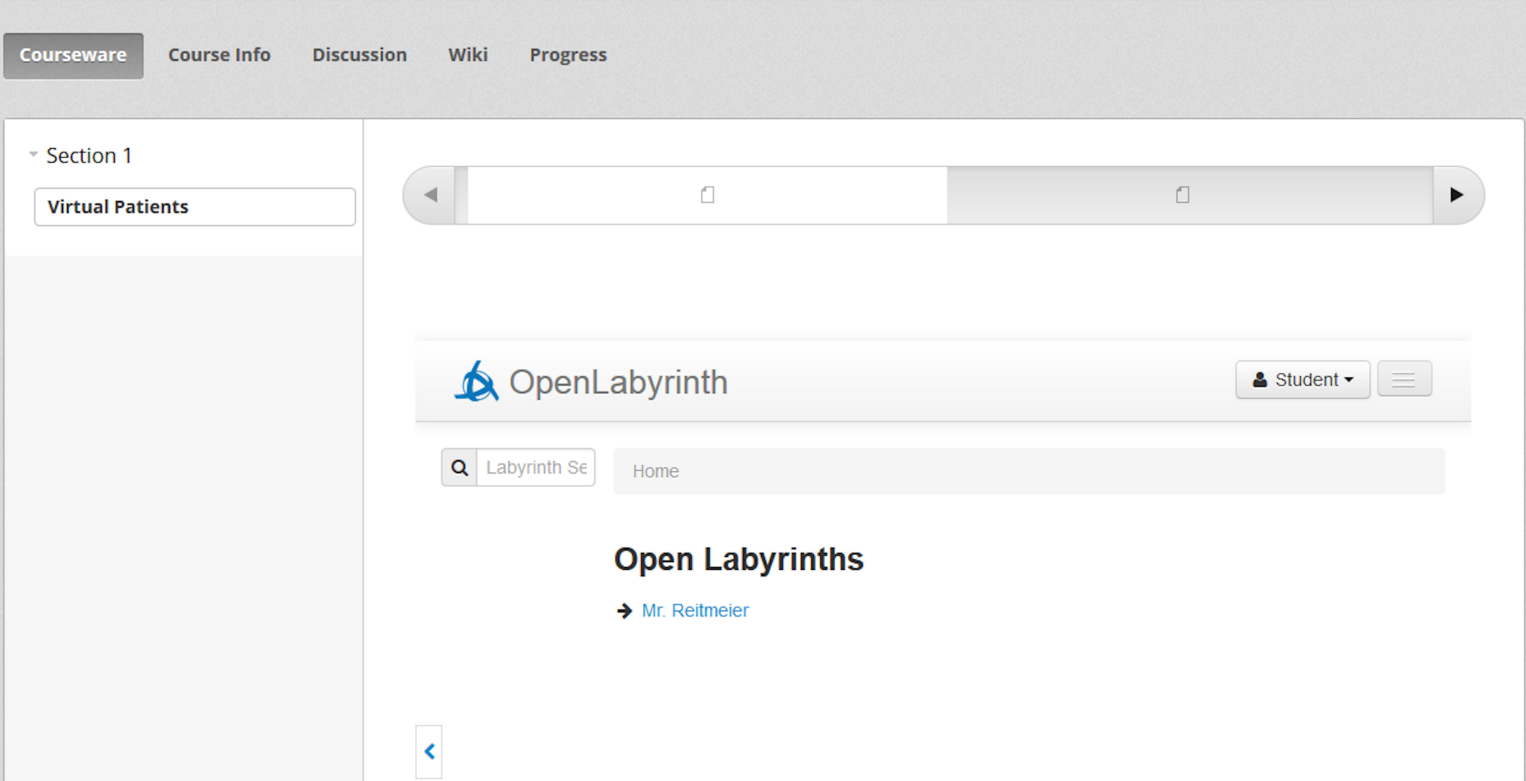
A learner is authorized in Open Labyrinth to view the learning content.

**Figure 6:**
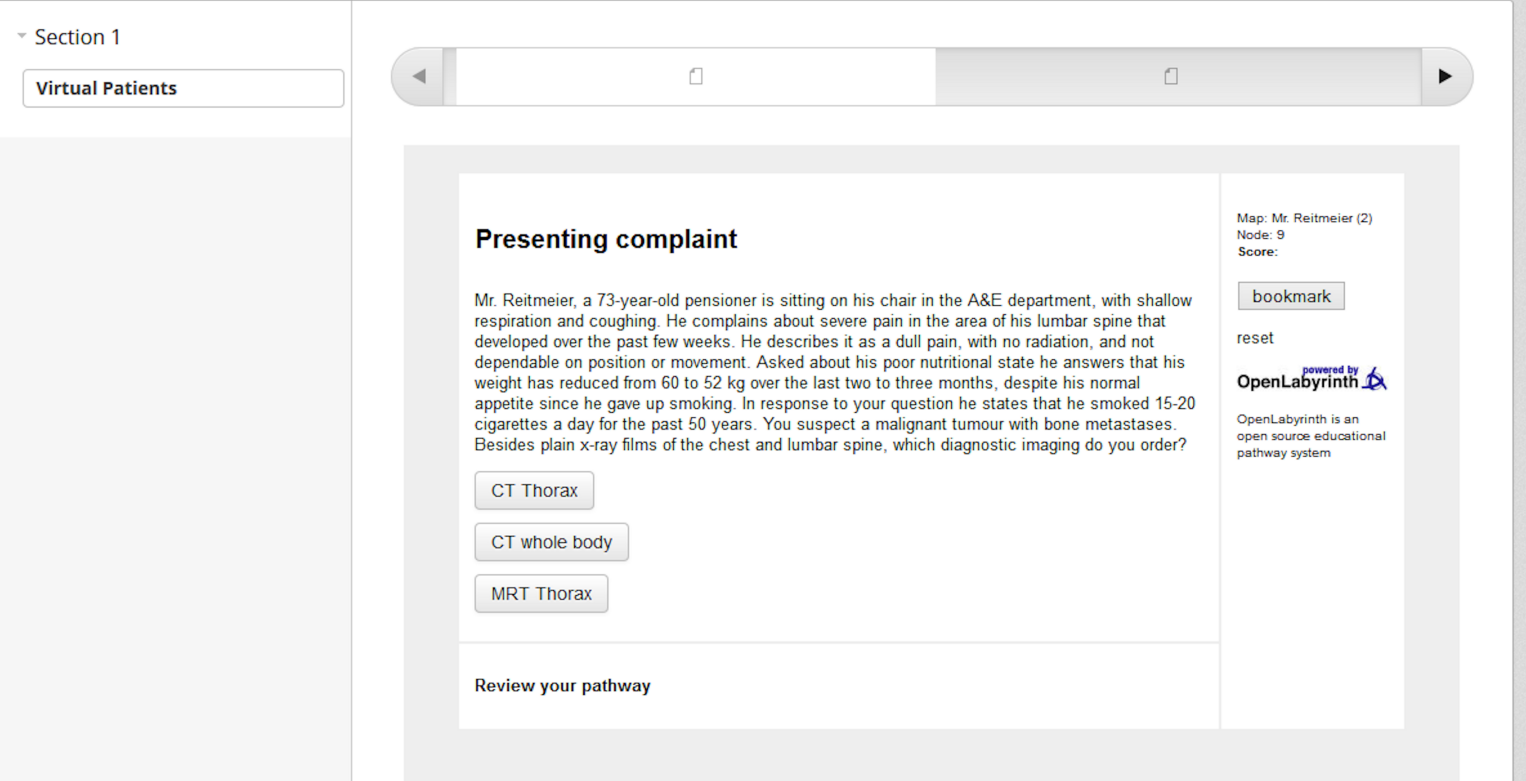
A learner accesses the VP case.

The following pictures depict the learner’s perspective while accessing the content by getting authorized in Open Labyrinth (Fig. 5) and trying the VP case (Fig. 6).

For all the test cases we also verified that providing wrong credentials does not allow the user to enter the system.

## Discussion

### Discussion on the results

In this paper we explored the possibility of integrating MOOCs with VPs. Adding virtual patients to MOOCs provides the learner with possibilities for active, exploratory acquisition of competencies such as clinical reasoning. Integrating MOOCs with VPs was discussed in the past in the context of investigating the potential educational benefits of three previously proposed educational scenarios (Stathakarou, Zary & Kononowicz, 2014). However, a practical implementation of selected technical elements was reported for the first time in this study. The challenge involved the selection of a suitable standard and extending the source code of a spacious open-source project in several places. This implementation enables further research on the educational benefits of the integration as well as their evaluation.

The selected technical requirement to be addressed was the SSO; a common requirement in the three use cases. The planned objectives of the study have been reached; Open Labyrinth has been integrated into the OpenEdx platform as an example of integrating VP systems with MOOC platforms.

The SSO was achieved by the use of the BLTI standard. The implementation was verified by the use of two test cases that were created with the aim of demonstrating a transparent authorization process for the two different types of users. In particular, the users logged into the OpenEdx platform may access the content in the integrated Open Labyrinth without repeated entry of their credentials. The instructors from OpenEdx get automatically authorized by Open Labyrinth and may view, edit, delete, or author a new VP by acquiring the corresponding administrative user rights to the Open Labyrinth VP system. The learners from OpenEdx get authorized in Open Labyrinth and may view the VP cases.

### Limitations and future studies

From the identified technical requirements we isolated and implemented just one for the prototyping. The approach to addressing this requirement was guided by a selected e-learning standard. The verification of the pilot implementation was based on two proposed test cases, the nature of which was determined by the aim of the study. In particular, the test cases were based on a pilot course in OpenEdx and a single inserted VP case. Hence, evaluating the user experience was not included in the objectives of this study. Future studies may investigate more advanced tests while exploring the user experience.

The integration demonstrated in the current study was based on the example of a single VP system and a MOOC platform: even though there are no reasons to suspect that the selected platforms were non-representative, future studies may investigate the integration strategies in a wider perspective including different VP and MOOC systems as well as achieving a tighter integration of the system by addressing the remaining identified requirements.

For the sake of simplicity of the implementation, we extended manually Open Labyrinth’s database, in the particular context of Open Labyrinth and OpenEdx systems, to include the tool consumer’s (OpenEdx) id and credentials. Other tool consumers may be added manually in the database to allow the integration of Open Labyrinth. However, this functionality could be automatized by enabling tool consumers to add appropriate credentials (key, secret) using a dedicated graphical user interface. This would require a careful design to ensure the security of the consumers’ credentials and the users’ information during the control process. Moreover, Open Labyrinth should be modified in order to accept and store potential extra parameters transmitted by the launch messages, since the set of parameters may differ between the consumers.

## Conclusion

The emergence of MOOC technology provides new opportunities to support the learning process. However, their current form is limited to the passive transmission of knowledge, based mainly on video-based lectures combined with self-assessment questions. Moreover, their application in healthcare education is still in early stages of investigation. This study demonstrated that extending MOOCs in order to support healthcare education can be achieved by integrating domain specific software.

In this paper we investigated the technical perspective of integrating VPs in MOOCs, aiming to set a base for future investigation of the topic; the pilot implementation provides evidence about the potential of integrating VP systems with MOOC platforms on the example of a transparent authentication mechanism, inviting further research for a complete integration and implementation of the suggested use cases.

## List of abbreviations

EC2: Amazon Elastic Compute Cloud
BLTI: IMS-Basic LTI Standard
CME: Continuing Medical Education
eViP: Electronic Virtual Patient project
MIT: Massachusetts Institute of Technology
MVP: MedBiquitous Virtual Patient standard
MOOCs: Massive Open Online Courses
SSO: Single-Sign On mechanism
SPOCS: Small Private Online Courses
VLEs: Virtual Learning Environments
VPs: Virtual Patients
